# The Global Challenge to Prevent Breast Cancer: Surfacing New Ideas to Accelerate Prevention Research

**DOI:** 10.3390/ijerph17041394

**Published:** 2020-02-21

**Authors:** Nicholas J. Anthis, Marion H. E. Kavanaugh-Lynch

**Affiliations:** California Breast Cancer Research Program, University of California Office of the President, 300 Lakeside Drive, 6th Floor, Oakland, CA 9461, USA; marion.kavanaugh-lynch@ucop.edu

**Keywords:** breast cancer, cancer research, primary prevention, challenge prize, innovation

## Abstract

Despite increases in screening and advances in treatment, breast cancer continues to be the most common cancer and cause of cancer deaths among women worldwide, and breast cancer rates have remained steady for decades. A new focus on population-level primary prevention is needed to tackle this disease at the most fundamental level. Unfortunately, only a small fraction of breast cancer research funds currently go to prevention. The California Breast Cancer Research Program (CBCRP) seeks to change this. In order to accelerate breast cancer primary prevention efforts, in 2018, CBCRP launched the Global Challenge to Prevent Breast Cancer, a prize competition to foster and disseminate new and innovative prevention research ideas. This Special Issue highlights the results of the Global Challenge and other CBCRP primary prevention efforts.

## 1. Introduction

Breast cancer is the most common cancer in women and the largest cause of cancer deaths among women worldwide: there were an estimated 2.1 million new cases and 626,679 deaths in 2018 [1]. A woman in the U.S. has a 13% chance of being diagnosed with breast cancer at some point in her lifetime and a 2.6% chance of dying from breast cancer [2]. Addressing breast cancer is a multi-front effort across the cancer control continuum, from prevention to treatment to survivorship. Great strides have been made in therapies and standards of care, leading to decreased mortality in developed countries. However, breast cancer incidence has remained essentially unchanged for the last three decades [2,3,4,5], indicating that a fresh approach to preventing breast cancer across the population is needed.

Prevention efforts can be divided into primary prevention (preventing cancer before it starts) vs. secondary prevention (screening and early detection), as well as population-level prevention vs. targeted prevention. While widespread screening through mammography promised a shift from more-advanced disease to less-advanced disease at diagnosis, the increase in less-advanced disease has actually come from more diagnoses of presumed indolent disease rather than fewer diagnoses of breast cancer at an advanced stage, and the rate of diagnosis of advanced (late-stage) breast cancer has not declined significantly [3]. Given that adjuvant chemotherapy and radiation treatment has become the standard of care for most non-metastatic breast cancers, this high rate of diagnosis has a large fiscal and human cost for the health system and for every person subjected to surgery, radiation, and adjuvant hormonal or chemotherapy [6,7]. On the other hand, primary prevention holds the promise of avoiding these pitfalls and decreasing the incidence of both advanced and less advanced disease.

Prevention efforts can focus on individuals at higher risk (e.g., individuals with BRCA gene alleles associated with a higher risk of breast cancer) or on the population at large. Although targeting the high end of the risk spectrum can be impactful for those individuals, it still fails to prevent the vast majority of breast cancer cases, which fall in the “normal” range of the bell curve of breast cancer risk. Additionally, for most people, we cannot precisely identify their breast cancer risk [8], and most cases of breast cancer are sporadic and not linked to any known risk factors [9]. Population-level prevention instead aims to shift the whole curve toward lower risk. Instead of looking at individual risk, if we look at risk across the population, we can identify and remove those common factors leading to a higher breast cancer incidence across the population (e.g., limiting exposures to environmental carcinogens and endocrine-disrupting compounds to which the population in general is exposed) [10]. The most impactful public health interventions are foundational efforts at system-level change across the population, addressing social determinants of health, and providing the context for individuals to lead healthier lives [11]. Such efforts have been effective in reducing tobacco use, especially in California, and thus markedly reducing the incidence of tobacco-associated cancers [12]. Finally, the consistently wide variation in breast cancer incidence rates across the world and the changes in incidence that occur when individuals migrate from low-incidence countries to high-incidence countries demonstrate the cancer preventive impact of changing our environment [13]. Thus, population-level primary prevention is an area of great research need and potential.

Yet, we have not risen to this challenge. Of the roughly USD 1 billion or more spent on breast cancer research each year across the globe, only a small fraction (5%–6%) is dedicated to prevention [14]. This trend is consistent across time, geography, and cancer type. For example, in the U.S., the National Cancer Institute (NCI) devoted 5.7% of its 2018 budget to “cancer prevention and control” [15]. Why are efforts to lower the incidence of breast cancer so poorly resourced? Reasons for this could include the lack of commercial incentives for developing public health interventions or that these efforts are not aligned with recent research interests and trends focused on precision medicine and molecular solutions. Another possibility is that the field needs new ideas and motivation to pursue these vexing research challenges. The recent Interagency Breast Cancer Environmental Research Coordinating Committee (IBCERCC) report, *Breast Cancer and the Environment: Prioritizing Prevention*, recommends that transdisciplinary approaches and targeted studies are needed, as well as a larger focus on the chemical and physical environment [16]. Our own experience at the California Breast Cancer Research Program is that calls for research proposals in primary prevention go largely unanswered, and our own efforts to engage experts in developing specific projects in primary prevention have been disappointing. Finally, knowledge must be translated into effective education, interventions, and policy change. Colditz et al. write: “Knowledge of the causes of cancer does not guarantee that action will be taken to reduce exposure to carcinogenic factors or to increase preventive behaviours. Despite accumulating evidence that indicates that most cancer cases could be prevented, few national prevention efforts have been launched.” [17].

This Special Issue seeks to address this gap by highlighting cutting-edge ideas for future research on methods of prevention of breast cancer through social and environmental interventions. Each manuscript in this Special Issue examines an innovative method by which we could alter the trajectory of social and environmental exposures that impact the population incidence of breast cancer. Six of these papers stem from the Global Challenge to Prevent Breast Cancer that the California Breast Cancer Research Program (CBCRP) launched in 2018. Another paper covers the results of an earlier project CBCRP funded to develop a Comprehensive Breast Cancer Primary Prevention Plan for California. A common theme across these articles is a focus on the *primary* prevention of breast cancer, especially at a population level.

## 2. Why a Global Challenge to Prevent Breast Cancer?

Prevention is a strategic priority for CBCRP, yet the program has historically received a dearth of proposals in this area. CBCRP was established in 1993 and is the largest state-funded breast cancer research effort in the U.S. After about a decade of funding investigator-initiated awards, in 2004, CBCRP launched its Special Research Initiatives, devoting 30% of research funds to research on environmental causes of breast cancer and the unequal burden of the disease. Over the course of three years, CBCRP released nine highly targeted funding opportunities that were developed by a panel of outside experts, and we funded USD 20.5 million across 26 awards.

In 2010, CBCRP launched its second round of Program Initiatives, adding primary prevention as well as population-level interventions and targeted interventions for high-risk individuals and devoting 50% of its research funds to these priority areas [18]. Fifteen targeted funding opportunities have been released or planned for release. To date, USD 18.8 million has been spent on 20 awards (two more funding opportunities are in progress). However, of these, only USD 1.3 million was spent on two awards focused primarily on population-level prevention, including the Primary Prevention Plan project highlighted in this Special Issue.

In 2015, CBCRP launched its third round of Program Initiatives, continuing the focus on environmental contributors, health disparities, and population-level prevention as focus areas. As part of this effort, we sought innovative solutions to garner more cutting-edge fundable prevention research proposals. We hypothesized that the lack of proposals received in this area in the past was due in part to a lack of ideas that researchers were excited to pursue, so we focused on generating new ideas. CBCRP devised the Global Challenge to Prevent Breast Cancer to crowdsource new ideas on breast cancer primary prevention research in order to identify exciting new directions where CBCRP’s funding could make the greatest difference and accelerate progress in the field more broadly. We launched the Global Challenge to look for both exciting new prevention research ideas that CBCRP could pursue and ideas that could inspire other funding agencies, researchers, and the general public to make prevention a priority.

## 3. Designing and Preparing for the Global Challenge

New to CBCRP’s third round of Program Initiatives was the idea that one source of research ideas could be a crowdsourced idea-generating competition or “grand challenge”. Challenges and incentive or inducement prizes are powerful tools for solving otherwise intractable problems and effecting change [19,20,21,22]. They are an approach that has been adopted by many philanthropies and governments, especially within the last decade. During the Obama administration, the U.S. federal government greatly expanded its use of challenges, including the creation of Challenge.gov [23]. One can tailor the design of a competition and amount of the prize to achieve a range of outcomes. Examples range from the National Eye Institute’s Audacious Goals challenge [24], which offered several USD 3000 prizes for ideas on new goals for the institute’s work on vision research—to the Ansari X Prize [25]—which offered one USD 10 million prize for the development and demonstration of a “reliable, reusable, privately financed, manned spaceship”. The Global Challenge to Prevent Breast Cancer was an example of an ideation challenge, a competition tailored to generate new ideas.

The literature provides various examples and best practices for how to run a challenge competition [19,20,21,22]. Key stages in the process were confirming that a challenge was the best mechanism for our needs and then scoping, designing, and planning the competition [20]. We chose to proceed with the challenge in part because we believed that crowdsourcing from a broad, global pool of innovators would lead to novel ideas more likely to shake up the status quo. We also identified the importance of generating ideas from both the scientific and lay/advocacy communities. As we scoped and designed the challenge, we sought input from various stakeholders, including scientists/researchers and advocates/non-researchers. We held round-table discussions with subject matter experts, and we designed a survey that we sent to previous advisors. The input we received from this survey proved invaluable in terms of helping scope the challenge, revealing barriers to entry, prioritizing motivators for participation, and identifying methods for outreach.

The survey revealed a few key potential barriers to entry. One concern that was common among researchers regarded intellectual property (IP) and the idea of giving up a potential research idea. We took a few steps to mitigate this concern. Ideas were submitted to the challenge under a Creative Commons Attribution (CC BY) license, meaning that when an applicant submitted their idea, they would be allowing the challenge organizers to further develop the idea—with attribution to the original applicant—and the applicant could still pursue the idea as well. We also emphasized that challenge winners would be able to stay involved with the idea and see it through to fruition. Winners whose idea was selected for further development would be invited to work with CBCRP to craft a concept paper based on the idea that will form the basis for a research funding opportunity and then have the opportunity to serve as a consultant to teams that receive grants. Researchers based in California or collaborating with a California-based scientist could also submit a proposal in response to any funding opportunity developed from the challenge (winners would have to choose between one of these avenues for involvement: either developing the idea with CBCRP or applying for funding, but not both). Non-researchers raised concerns about competing directly against scientific experts. This concern was addressed by creating two separate grand prizes: one reserved for an advocate, patient, consumer, activist, layperson, or anyone else who is not a researcher or scientist and the other for a researcher. Both researchers and advocates emphasized a desire to team up with others, so instead of only opening the competition to individuals, we allowed teams to participate as well.

The survey also gave us a window into the motivators that might incentivize someone to participate in the challenge. For both researchers and non-researchers, the most highly rated incentive was the knowledge that they may be playing a role in helping prevent breast cancer in the future. The opportunity to influence the direction of the field and how research funds are spent was also a strong motivator. The more tangible motivators were also similar between researchers and non-researchers, though their relative value differed somewhat. For researchers, the highest rated tangible incentive was the opportunity to publish their idea in a well-respected journal. For non-researchers, it was the invitation to attend an in-person event and meet leaders in the field. Both groups also identified the opportunity to present their idea to leaders in the field and receive feedback from prominent researchers and advocates in the field as at least somewhat motivating. Interestingly, the cash prize was not identified as a significant motivator among either group. Only about half of respondents agreed that a USD 5000 prize would incentivize them to participate. Answers about what prize value would incentivize them varied widely, from 0 to USD 100,000. Therefore, we set the prize at USD 5000, which Goldhammer et al. had identified as the median value for a first-place prize in challenges designed to attract new ideas [19]. Given these results, we emphasized both the tangible and intangible aspects of the competition, especially that participants could watch their “impossible” idea become possible and be a part of it when it did.

## 4. Launching and Promoting the Global Challenge

We launched the Global Challenge to Prevent Breast Cancer on 21 September 2018. The challenge was a signature commitment to the Biden Cancer Initiative’s effort to double the rate of progress against cancer, and our launch coincided with the Biden Cancer Summit held that day. The launch of the challenge coincided with the next phase of the outreach campaign—comprising partner outreach, email, and social media—which continued through the application deadline. The challenge literature emphasizes the importance of partnerships [19,20,21,22]. Early in the challenge process, CBCRP contracted with the Consensus Building Institute (CBI) to serve as the scientific convener for our third round of Program Initiatives, and CBI became a partner in planning and carrying out the Global Challenge. We were fortunate to have the assistance of many other agencies in spreading the word through their own communication channels.

**Figure 1:**
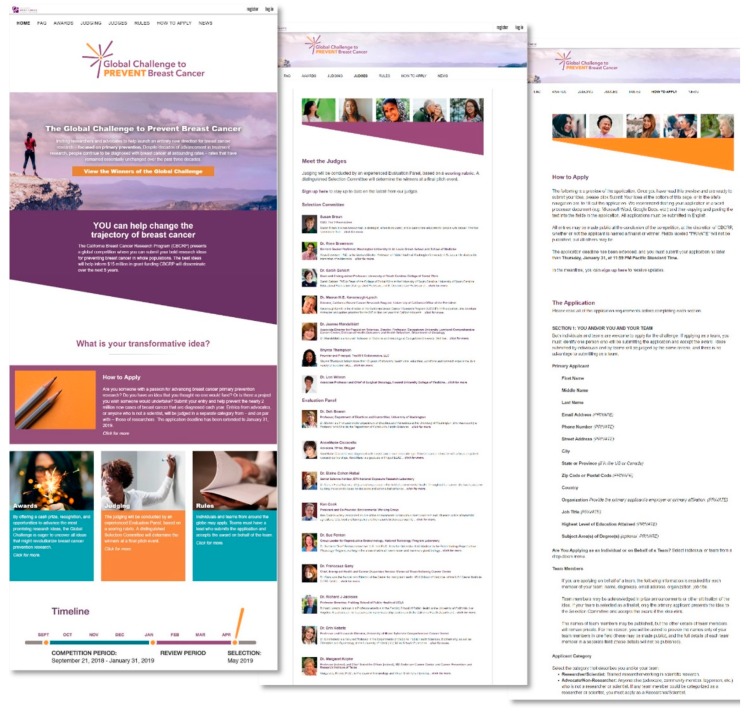
Screenshots of the Global Challenge to Prevent Breast Cancer website, showing portions of the homepage, judges page, and application instructions.

The web platform used for the challenge was IdeaScale, billed as an “idea management platform”. On IdeaScale, we built a public-facing website with information and news about the challenge, an application form, and a system to run the review process (Figure 1).

The Global Challenge website laid out the scope of the challenge, noting that we sought breakthrough ideas to catalyze the field of breast cancer prevention research. Submissions needed to fulfill the following criteria:

- Address *primary* prevention of breast cancer (preventing breast cancer before it occurs);
- Focus on prevention, not just understanding the causes of breast cancer;
- Be aimed at reducing breast cancer in whole populations, not just in groups at highest risk; and
- Be implementable in California and able to be advanced in a significant way within five years.

Individuals and teams were invited to apply online. The competition was open to anyone across the globe, and there were separate categories for researchers/scientists and advocates/non-researchers. The application was kept short to not be a barrier to entry. The main part of the application was a description of the idea, limited to 1000 words or less. This statement was supposed to describe five aspects: the specific problem that the idea aims to solve; the proposed research and how it would make meaningful progress within five years; the resources needed to solve the problem; how the idea is innovative; and how the idea would accelerate progress in breast cancer prevention.

Before we launched the Global Challenge, we laid the foundation for promoting it. This included developing outreach content including an online toolkit, which was created in consultation with a communications firm. We also cultivated a broad network of partners, including members of the International Cancer Research Partnership (ICRP). Although previous attempts to recruit co-sponsors for the challenge had been unsuccessful, we were able to build a network of dozens of partner organizations to help spread the word. This was especially important because being a *global* challenge, we wanted to solicit ideas from across the world and outside of our program’s main audience, which is in California. By engaging with an international network of funding organizations, cancer advocacy organizations, and others, we were well placed to reach that audience. Before launch, we also recruited a judging panel of highly respected experts and advocates, who could also help with the promotion of the challenge

When the final application deadline was reached on 31 January 2019, we had received 46 submissions from 12 countries across the globe. Summaries of these entries can be viewed online at http://cabreastcancer.org/global-challenge/ideas.html.

## 5. Selecting Winning Ideas

Applications to the Global Challenge were evaluated through a two-stage process, which was designed based on CBCRP’s extensive grantmaking experience. First, all applications were judged by members of an evaluation panel, composed of respected researchers and advocates. After all the applications were judged, the evaluation panel met to select the 10 finalists. The finalists were then invited to present their research ideas to the selection committee at a public event. The selection committee was composed of respected researchers and advocates who were serving on CBCRP’s Program Initiatives Steering Committee. In each stage of the review, the panel was asked to evaluate the idea on three criteria: boldness, impact, and relevance.

In the first stage of review, the evaluation panel reviewed the written submissions. In the second stage, finalists gave brief “flash talk” presentations before the selection committee. The reviewers were asked to review the substance of the underlying idea, not the quality of the presentation. A key concern was instilling in the reviewers that the Global Challenge was different from the typical grant review panel with which they might be much more familiar. It was stressed that we were interested in all ideas that might advance breast cancer prevention. The Global Challenge was different from a grant competition in that applicants were submitting ideas that may be much more preliminary—and which they may not even be interested in or capable of carrying out themselves. We asked reviewers to be open minded.

**Figure 2:**
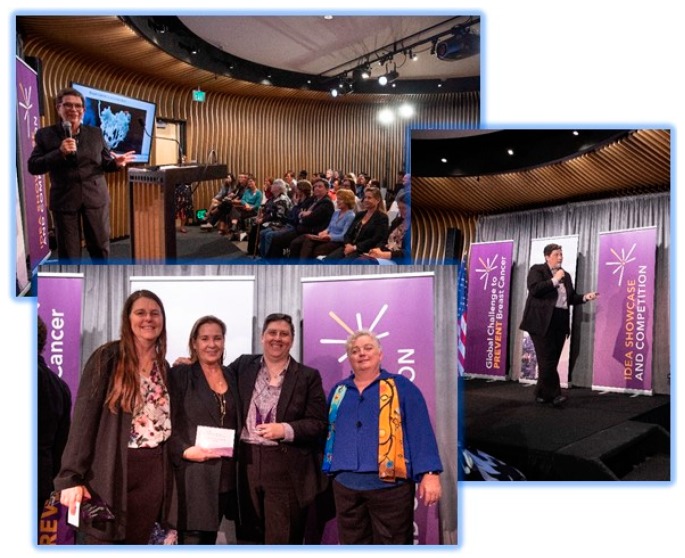
Photos taken at the Global Challenge Idea Showcase and Competition event. Top left: Keynote address by Susan M. Love. Bottom left: California Breast Cancer Research Program (CBCRP) Director Marion H.E. Kavanaugh-Lynch (right) poses with challenge winners Victoria Seewaldt (left), Michele Atlan (middle), and Nancy Buermeyer (right). Right: Nancy Buermeyer (grand prize, advocate) presents her idea. (Photo credits: Michael Pegram).

After the first stage of review, the 10 finalists were invited to present their ideas at a public event. The Global Challenge Idea Showcase and Competition was held on 15 May 2019, before a live audience in San Francisco, California, and was also streamed online to a global audience (Figure 2). Each finalist was given the opportunity to present a five-minute flash talk on their idea. Strict guidelines were given in terms of content, organization, and use of slides. To enhance the presentations, each finalist was given a thirty-minute speaker training session.

We had a combined in-person and online audience of more than 350 people. The judges scored each presentation on each of the three criteria after each talk, and they used those results to choose the two grand prize winners. In addition, audience members were able to vote online during the keynote presentation, and the top vote-getter was named the audience choice winner. The winners and finalists are listed in Table 1. Videos of the Global Challenge Idea Showcase and Competition can be viewed at http://cabreastcancer.org/global-challenge/video.html.

## 6. Lessons Learned and Next Steps: Turning Ideas into Research

Running an idea-generating challenge is only the first step in the process of driving change [19]. The next phase of work involves feeding Global Challenge ideas into our Program Initiatives process, developing the top ideas submitted to the challenge into research funding opportunities. Through our Program Initiatives, CBCRP has engaged an external steering committee and a collection of strategy advisors to identify and prioritize research questions based on challenge entries. The highest priority research questions, which may include ideas from the challenge, will be fleshed out into concept papers, which CBCRP will develop into funding opportunities. Investigators in California will then be able to apply for research funding in these areas, and their applications will be evaluated through CBCRP’s competitive, National Institutes of Health (NIH)-approved scientific review process. The end result is that CBCRP will fund research on high-priority topics that would have otherwise gone unfunded.

Over the course of the Global Challenge, many lessons were learned. A key takeaway is how important a network of partners is to get the word out, especially for a global competition. Outreach ended up being one of the largest challenges; we did not receive as many applications as we sought, and not all of the applications were on target. This underlines the importance of having clear expectations and guidelines set up from the beginning. We also had issues with the technology platform that we were using for the challenge, and it would have been better to have spent more time up front to ensure that the platform was a good fit for the competition we wanted to run.

## 7. Bold Ideas for Breast Cancer Prevention Research

The Global Challenge received submissions from researchers/scientists and advocates/non-researchers from across the globe. The ideas covered a broad range and diversity of topics, from environmental exposures to education. All 10 finalists in the Global Challenge were invited to submit a paper to this Special Issue describing their idea, and we are excited to present six of these ideas here. We also present a paper about the CBCRP-funded project to create a Comprehensive Breast Cancer Primary Prevention Plan for California (see Table 1 for details).

We hope that these ideas will catalyze other breast cancer prevention research efforts across the globe and help prevent the more than 2 million cases [1] of breast cancer that occur each year. We hope they will ignite the imagination and inspire others to prioritize and invest in prevention research. Prevention should be the next horizon for breast cancer research, and we aim to catalyze it in part by presenting the exciting ideas in this Special Issue.

## Figures and Tables

**Table 1:** The winners and finalists of the Global Challenge to Prevent Breast Cancer and articles published in this Special Issue.

Status	Winner/Finalist	Co-Applicants/Co-Authors	Article/Idea Title	Ref.
Grand Prize (Researcher)	Victoria L. Seewaldt	Rama Natarajan et al.; the SoCal STEM and Community Outreach Team	Environmental Exposures during Puberty: Window of Breast Cancer Risk and Epigenetic Damage	[26]
Grand Prize (Advocate)	Nancy Buermeyer	Janet Nudelman	California Ports: Air Pollution Interventions and Breast Cancer Risk in Local Communities	
Audience Choice Award	Michele Atlan	Josh Neman	Targeted Transdermal Delivery of Curcumin for Breast Cancer Prevention	[27]
Finalist	Vincent Bessonneau	Ruthann A. Rudel	Mapping the Human Exposome to Uncover the Causes of Breast Cancer	[28]
Finalist	Gertrude C. Buehring	Hannah M. Sans	Breast Cancer Gone Viral? Review of Possible Role of Bovine Leukemia Virus in Breast Cancer and Related Opportunities for Cancer Prevention	[29]
Finalist	Barbara A. Cohn	Mary Beth Terry	Environmental Influences on Mammographic Breast Density in California: A Strategy to Reduce Breast Cancer Risk	[30]
Finalist	Hannah Lui Park		Epigenetic Biomarkers for Environmental Exposures and Personalized Breast Cancer Prevention	[31]
Finalist	Andrea R. Hindman	Jessica S. Helm	Keeping Abreast of Prevention in Chemical Safety Testing	
Finalist	Laura Markuly		Low Dose Naltrexone (LDN): The New Breast Cancer Prevention	
Finalist	Thea D. Tlsty		The Mother of All Primary Prevention Assays	
N/A ^1^		Mary C. White, et al.	An Expanded Agenda for the Primary Prevention of Breast Cancer: Charting a Course for the Future	[32]

^1^ The article by White et al. was not based on a Global Challenge entry but instead describes another primary prevention effort sponsored by CBCRP.
